# Exocrine Pancreatic Insufficiency in Diabetic Patients: Prevalence, Mechanisms, and Treatment

**DOI:** 10.1155/2015/595649

**Published:** 2015-03-29

**Authors:** Matteo Piciucchi, Gabriele Capurso, Livia Archibugi, Martina Maria Delle Fave, Marina Capasso, Gianfranco Delle Fave

**Affiliations:** Digestive and Liver Disease Unit, S. Andrea Hospital, Faculty of Medicine and Psychology, Sapienza University of Rome, 00189 Rome, Italy

## Abstract

Pancreas is a doubled-entity organ, with both an exocrine and an endocrine component, reciprocally interacting in a composed system whose function is relevant for digestion, absorption, and homeostasis of nutrients. Thus, it is not surprising that disorders of the exocrine pancreas also affect the endocrine system and vice versa. It is well-known that patients with chronic pancreatitis develop a peculiar form of diabetes (type III), caused by destruction and fibrotic injury of islet cells. However, less is known on the influence of diabetes on pancreatic exocrine function. Pancreatic exocrine insufficiency (PEI) has been reported to be common in diabetics, with a prevalence widely ranging, in different studies, in both type I (25–74%) and type II (28–54%) diabetes. A long disease duration, high insulin requirement, and poor glycemic control seem to be risk factors for PEI occurrence. The impact of pancreatic exocrine replacement therapy on glycemic, insulin, and incretins profiles has not been fully elucidated. The present paper is aimed at reviewing published studies investigating the prevalence of PEI in diabetic patients and factors associated with its occurrence.

## 1. Introduction

Pancreas is a doubled-entity organ, with both an exocrine and an endocrine component, reciprocally interacting and closely cooperating for the digestion, absorption, and metabolism of oral nutrients [1].

Thanks to its lobulated tubulo-alveolar-acinar structure, endocrine islet cells secrete their hormones in the insulo-acinar portal vascular pathway, thus regulating and conditioning ductal and acinar cell exocrine activities [2].

It has been recently demonstrated that the rise of insulin and other pancreatic islet peptides (amyline, C-peptide, and urocortin-III), following the postprandial glucose rise, stimulates the exocrine pancreatic function [3]. On the other hand, the increase of glucagon during fasting depresses pancreatic enzyme and juice secretion.

Ductal and acinar cells, in turn, affect the physiology of endocrine islet cells through cytokines and growth factor secretion [2, 3].

Furthermore, the endocrine cells disseminated in the small bowel secrete two peptide hormones, glucagon-like peptide-1 (GLP-1) and glucose-dependent insulinotropic hormone (GIP), known as incretins, that are released soon after lipid and carbohydrate ingestion, thus stimulating a strong insulinic production. A deregulation of incretin secretion is one of the causes of the altered insulinic response in type II diabetes [4].

With regard to chronic pancreatitis patients, it is well described that they may suffer from a peculiar form of diabetes (type III diabetes), characterized by the destruction of islet cells by inflammatory and fibrotic injury. Type III diabetes differs from type I diabetes as the damage of pancreatic islets is not limited to insulin secreting beta cells but is more diffused as it also affects glucagon and pancreatic polypeptide secreting alpha and PP cells. In addition to this, in type III diabetes the malabsorption of nutrients due to pancreatic exocrine insufficiency (PEI) results in impaired incretin secretion with consequently diminished insulin release. Furthermore, as suggested by the 2012 PancreasFest recommendations, a quote of type III diabetes patients may also have preexisting risk factors for type II diabetes (e.g., insulin resistance, obesity, or dietary habits) that further complicate the optimal regulation of glucose metabolism resulting in a severe disease with large swings in blood sugar that are hardly controlled [5].

On the other hand, less is known on the influence of diabetes on pancreatic exocrine function.

As the prevalence of diabetes in the general population is much higher than that of chronic pancreatitis, this relationship is potentially of great clinical relevance.

In the sixties, a few pioneering studies investigated exocrine pancreatic function in small series of diabetic patients, throughout direct duodenal juice collection (secretin cerulein test or SCT) and reported changes in both output and HCO_3_ concentrations [6–8].

More recently, thanks also to the availability of a noninvasive test for pancreatic exocrine function, such as measurement of fecal elastase, several studies aimed at investigating the prevalence of PEI in large cohorts of diabetic patients.

The present paper is aimed at reviewing findings of published studies investigating the prevalence of PEI in diabetic patients and possible risk factors and mechanisms associated with its occurrence and severity. The cases of patients with type I and type II diabetes will be discussed separately, in view of possible different features and pathogenic mechanisms.

## 2. Pancreatic Exocrine Insufficiency in Type I Diabetes

Abnormalities in histological features and imaging (MRI, CT, and ultrasound) of the pancreas of diabetic patients have been reported, as well as atrophy, fibrosis, changes in size, and morphology; several and various hypotheses have been proposed to explain these phenomena implicated in PEI occurrence in diabetics [9, 22–25].

The damage of the exocrine cells in type I diabetes is most likely multifactorial, with a number of possible causes: (a) the lack of the trophic action of insulin (and possibly of glucagon and somatostatin) on acinar cells; (b) an involvement of the exocrine tissue in the autoimmune destruction of islet cells; (c) autonomic diabetic neuropathy leading to enteropancreatic reflex impairment; (d) hypoxic sufferance of exocrine tissue due to microvascular damage [26, 27].

In this view, type I diabetes, which is linked to a primary autoimmune process and is characterized by early occurrence, severe insulin deficiency, and long standing disease, with a high rate of neural and vascular complications, seems to be more frequently associated with PEI than type II.

PEI seems to occur early in type I diabetes patients, and 2 studies assessing fecal elastase concentrations in children and young patients, ranging from 2 to 25 years of age, detected severe (defined by a cut-off of fecal elastase <100 *μ*g/g) to moderate PEI (elastase <200 *μ*g/g) in 10% to 45% of screened subjects [28, 29].

In adult series of type I diabetes patients, the prevalence of both severe (10–30%) and moderate (22–56%) PEI seems higher than in children, possibly suggesting that exocrine pancreatic function decreases in parallel with the duration of disease and the increase in insulin requirement. The largest study [10] investigating risk factors for PEI occurrence in 195 type I diabetes patients demonstrated a strong association between PEI and disease duration, but these results were not confirmed in other settings and no association with insulin requirement or elevated HbA1c was observed. However, these latter studies had several limitations, such as a smaller number of subjects included, not well-defined enrollment criteria, and poor investigation of risk factors for PEI occurrence. Studies evaluating pancreatic exocrine function in adult type I diabetes patients are summarized in Table 1 [9–15].

## 3. Pancreatic Exocrine Insufficiency in Type II Diabetes

Physiopathological mechanism involved in PEI occurrence in type II diabetes seems to be similar to that reported above for type I diabetes. In particular, in these subjects, without autoimmune damage and insulin deficiency, autonomic neuropathy and microvascular damage may play a key role in inducing pancreatic atrophy and fibrosis. Hayden and colleagues observed that type II diabetes human and rodent pancreas specimens show a loss of desmosomes and adherens junctions between islet and acinar cells, due to fibrosis and remodeling of the islet-acinar interface, that may result in an impaired function [3].

Regarding PEI prevalence in type II diabetes more data are available, although these are somehow more heterogeneous. Studies evaluating pancreatic exocrine function in adult patients with type II diabetes are summarized in Table 2 [10, 12, 13, 15–21].

In the largest prospective study, performed on 1231 diabetic patients, Hardt and colleagues demonstrated a prevalence of PEI of 35% in 697 patients with type II diabetes. However, the observed correlations with disease duration and insulin therapy in the general population of 1231 subjects were not confirmed in the subgroup analysis of type II diabetes patients. Notably, this study did not specifically exclude cases with previous history of pancreatic disease, thus leading to a possible bias [16].

Subsequent studies, enrolling a smaller number of patients, confirmed a prevalence of PEI of about 30–40% in type II diabetes. Although these results are often heterogeneous, most studies concord that the need of insulin therapy is associated with a higher prevalence and with the severity of PEI [10, 13, 15–21].

Early onset of type II diabetes and long disease duration as well as poor glycemic control (expressed in particular as elevated HbA1c) seem to be risk factors for the occurrence and severity of PEI, although their role has not been confirmed in all studies and results were not always statistically significant [10, 13, 15–21]. This supports the hypothesis that a long, complicated type II diabetes, with a higher degree of microvascular damage, pancreatic fibrosis, and autonomic neuropathy, is more probably associated with PEI occurrence.

Interestingly, in most reports an elevated BMI seems to be a protective factor for the occurrence of PEI occurrence. However, this is most likely a bias, as patients with severe PEI would show malabsorption and malnutrition and consequent reduced BMI [10, 13, 15, 17–21].

## 4. Conclusions

The reciprocal relationship between the endocrine and exocrine pancreatic function is an interesting and relatively poorly investigated area of research in which many questions still remain unanswered. Most studies investigating the prevalence of PEI in both type I and type II carry several limitations (summarized in Tables 1 and 2). All these mentioned studies have a “cross-sectional” design, and they only evaluated the prevalence of PEI and not its incidence over time in the course of the disease. One study prospectively evaluated PEI with SCT, although during a short and not standardized follow-up period and only in 17 type I diabetes patients. In contrast with previous observations, this latter study, with all the above mentioned limitations, observed no changes in the exocrine pancreatic function during the follow-up period [30].

The reported prevalence of PEI observed in type I (25–74%) and type II (28–54%) diabetes in different studies assessing PEI with fecal elastase measurement is also highly variable. This heterogeneous prevalence could be due to the low sensibility of fecal elastase, as reported by Hahn and colleagues in a small series of type I diabetes patients, who underwent both direct and indirect pancreatic function tests [14]. A recent study reported that a noninvasive 13C-Mixed Triglyceride Breath Test (C-MTGT) for evaluation of pancreatic exocrine function can detect mild to moderate PEI in diabetes mellitus. However, the specificity of C-MTGT, compared to direct SCT, is low in these patients, probably because nonpancreatic mechanisms contribute to decrease intestinal lipolysis [31]. To better assess PEI prevalence in diabetic patients, future studies should also take into account the dilution of elastase enzyme in stools during diarrhoea, which is, irrespectively to PEI, a symptom often present in diabetic subjects because of bacterial overgrowth and diabetes-induced vascular or neuropathic complications [14, 31].

As regards the aetiology of PEI, a long duration of disease, high insulin requirement, and poor glycemic control, expression of more severe disease seems to be associated with PEI occurrence and severity. In type II diabetes, a more severe form of disease is more frequently associated with systemic complications such as autonomic neuropathy and microvascular damage, determining fibrosis and atrophy of the pancreas, and loss of communication in the islet-acinar-ductal axis and gastroenteropancreatic system.

In type I diabetes, the primary reduction of insulin levels due to the autoimmune mediated damage of islet cells results in a decreased trophic action on the exocrine cells.

This combination of multiple factors may explain the observed higher prevalence of PEI observed in type I compared to type II diabetes (about 60% versus 30% of cases). However, the mechanisms causing PEI in diabetes still need to be elucidated.

Furthermore, it is unclear which diabetic patients would benefit from screening for PEI in clinical practice, and there are very limited data regarding the possible impact of pancreatic enzyme replacement therapy (PERT) on diabetic subjects with PEI.

In a small series of 8 patients affected by chronic pancreatitis with PEI, half of them with type III Diabetes, Knop et al. evaluated the effect of PERT on glucose, insulin, GIP, and GLP-1 profiles after meal. Interestingly, PERT improved the response of GIP, GLP-1, and insulin response after meal, but not the postprandial glucose profile [32].

One might, therefore, hypothesize that, also in diabetic patients with PEI, PERT might ameliorate the postprandial response of insulinotropic intestinal peptides and subsequently the insulin secretion. However, no studies have yet evaluated the impact of enzymatic replacement on insulin and incretin levels in response to meal, in these subjects.

Only one prospective study evaluated the effect of PERT on 39 insulin dependent diabetic (type I or II) patients with PEI (defined as fecal elastase I concentrations <100 *μ*g/g) compared to 41 similar patients receiving placebo. This study showed that PERT improved steatorrhea and malabsorption symptoms in comparison to the controls receiving placebo but had no impact on glucose profile, insulin requirement, and HbA1c levels [17]. However, this study was performed on a small number of patients for a short period of time (3 months). Furthermore, all patients with non-insulin-dependent diabetes and with moderate PEI (fecal elastase concentrations <200 *μ*g/g) were excluded, and insulin, glucagon, and incretin levels, that might be modified by the enzymatic treatment, were not assessed [17].

Future studies evaluating the influence of PERT on glycemic control and insulin requirement should enroll a larger and more homogeneous population of diabetic patients, with treatment enduring for a longer period of time, and should also possibly evaluate the relation between PERT and incretins profile. On the other hand, the potential effect of treatment with incretins on the exocrine pancreas is also a controversial area for further research, as the relation between the exocrine and the endocrine pancreas represents a major field of investigation [33].

## Figures and Tables

**Table 1:** Investigation of pancreatic exocrine insufficiency with fecal elastase in type I diabetes patients.

Study	Patients	PEI prevalence	Factors associated with PEI	Strengths	Limitations
Hardt et al. 2002 [9] (cross sectional-study)	323	Elastase <200 = 51% Elastase <100 = 28.5%	(i) Association with diabetes duration, early onset, and insulin usage (ii) No association with age and sex	(i) Large cohort of patients (ii) Inclusion and exclusion criteria well designed	(i) Cross-sectional study without prospective evaluation of patients (ii) Risk factors subgroup analysis not performed in DI and DII patients

Larger et al. 2012 [10] (cross-sectional study)	195	Elastase <200 = 34% Elastase <100 = 19%	(i) Association with diabetes duration (ii) No association with insulin dose, elevated BMI, age, and early age at onset	(i) Large cohort of patients (ii) Risk factors subgroup analysis performed	(i) Cross-sectional study without prospective evaluation of patients (ii) Patients with type III diabetes included (iii) Inclusion and exclusion criteria not well specified

Icks et al. 2001 [11] (cross-sectional study)	112	Elastase < 200 = 45.5% Elastase < 100 = 25.9%	(i) Association with male sex and age (ii) No association with diabetes duration, elevated BMI, early age at diabetes onset, and smoke or alcohol usage	(i) Specifically performed on type I diabetes patients (ii) Case-control study (iii) Risk factors specifically and extensively evaluated	(i) Relatively small number of patients (ii) Cross-sectional study without prospective evaluation of patients

Cavalot et al. 2006 [12] (cross-sectional study)	66	Elastase < 200 = 25.8% Elastase < 100 = 10.6%	(i) No association with age, diabetes duration, elevated HbA1c, early age at diabetes onset, and elevated BMI	(i) Specifically performed on type I diabetes patients (ii) Extensive analysis on PEI-related clinical symptoms	(i) Small number of patients (ii) Cross-sectional study without prospective evaluation of patients

Vujasinovic et al. 2013 [13] (cross-sectional study)	50	Elastase < 200 = 6% Elastase < 100 = 2%	(i) No association with diabetes duration	(i) Extensive analysis on PEI-related clinical symptoms (ii) MRI with MRCP performed in all diabetic patients with PEI	(i) Small number of patients (ii) Enrollment criteria not well-defined (iii) Cross-sectional study without prospective evaluation of patients defined (iv) Poor risk factors analysis

Hahn et al. 2008 [14] (cross-sectional study)	33	Elastase < 200 = 27.3%	(i) No association with sex, age, diabetes duration, elevated HbA1c, early age at diabetes onset, and elevated BMI	(i) All patients underwent both SCT and indirect tests to assess PEI	(i) Very small number of patients (ii) Cross-sectional study without prospective evaluation of patients defined

Hardt et al. 2000 [15] (cross-sectional study)	31	Elastase < 200 = 56.7% Elastase < 100 = 30%	(i) Association with GI symptoms (ii) No association with diabetes duration and alcohol usage		(i) Very small number of patients (ii) Enrollment criteria not well-defined (iii) Cross-sectional study without prospective evaluation of patients defined (iv) No subgroup analysis performed for DI and DII

PEI = pancreatic exocrine insufficiency.

MRI = Magnetic Resonance Imaging.

MRCP = Magnetic Resonance Cholangiopancreatography.

BMI = body mass index.

DI = type I diabetes; DII = type II diabetes.

**Table 2:** Investigation of pancreatic exocrine insufficiency with fecal elastase in type II diabetes patients.

Studies	Patients	PEI prevalence	Factors associated with PEI	Strengths	Limitations
Hardt et al. 2003 [16] (cross-sectional study)	697	Elastase < 200 = 35.9% Elastase < 100 = 19.9%	(i) Association with diabetes duration, early onset, and insulin usage (ii) No association with age and sex	(i) Large cohort of patients (ii) Inclusion and exclusion criteria well designed	(i) Cross-sectional study without prospective evaluation of patients (ii) Risk factors subgroup analysis not performed in DI and DII patients

Ewald et al. 2007 [17] (cross-sectional study)	546	Elastase < 100 = 21.1%	Not investigated	(i) Prospective evaluation of three months of pancreatic enzyme replacement therapy (ii) Well designed study (iii) Large cohort of patients	(i) Cross-sectional study without prospective evaluation of patients defined (ii) Risk factors analysis for PEI occurrence not performed

Rathmann et al. 2001 [18] (cross-sectional study)	544	Elastase < 200 = 30.3% Elastase < 100 = 11.9%	(i) Association with low BMI and elevated HbA1c (ii) No association with diabetes duration, insulin usage, age, sex, and smoke or alcohol usage	(i) Extensive analysis of risk factors and clinical features of patients (ii) Specifically performed on type II diabetes patients (iii) Large cohort of patients	(i) Cross-sectional study without prospective evaluation of patients

Larger et al. 2012 [10] (cross-sectional study)	472	Elastase < 200 = 20% Elastase < 100 = 12%	(i) Association with low BMI and insulin usage (ii) No association with diabetes duration and elevated HbA1c	(i) Large cohort of patients (ii) Risk factors subgroup analysis performed	(i) Cross-sectional study without prospective evaluation of patients (ii) Patients with type III diabetes included (iii) Inclusion and exclusion criteria not well specified

Vujasinovic et al. 2013 [13] (cross-sectional study)	100	Elastase < 200 = 5% Elastase < 100 = 3%	(i) No association with diabetes duration	(i) Extensive analysis on PEI-related clinical symptoms (ii) MRI with MRCP performed in all diabetic patients with PEI	(i) Small number of patients (ii) Enrollment criteria not well-defined (iii) Cross-sectional study without prospective evaluation of patients defined (iv) Poor risk factors analysis

Hardt et al. 2000 [15] (cross-sectional study)	83	Elastase < 200 = 46% Elastase < 100 = 16.9%	(i) Association with GI symptoms (ii) No association with diabetes duration and alcohol usage		(i) Very small number of patients (ii) Enrollment criteria not well-defined (iii) Cross-sectional study without prospective evaluation of patients defined (iv) No subgroup analysis performed for DI and DII

Mancilla A et al. 2006 [19] (cross-sectional study)	70	Elastase < 200 = 33% Elastase < 100 = 19%	(i) Association with diabetes duration (ii) No association with age, sex, elevated HbA1c, and insulin usage	(i) Specifically performed on DII patients	(i) Small number of patients (ii) Enrollment criteria not well-defined (iii) Cross-sectional study without prospective evaluation of patients defined (iv) Risk factors not well analyzed (v) Article available only in Spanish

Nunes et al. 2003 [20] (cross-sectional study)	42	Elastase < 200 = 36% Elastase < 100 = 22%	(i) No association with diabetes duration and insulin usage	(i) Case-control study (ii) Patients with PEI underwent US (iii) Specifically performed on DII patients	(i) Small number of patients (ii) Cross-sectional study without prospective evaluation of patients defined (iii) Risk factors analysis not extensively performed

Yilmaztepe et al. 2005 [21] (cross-sectional study)	32	Elastase < 200 = 28.1% Elastase < 100 = 3.1%	(i) No association with diabetes duration, sex, elevated HbA1c, alcohol usage, and elevated BMI	(i) Specifically performed on DII patients (ii) Case-control study	(i) Small number of patients (ii) Cross-sectional study without prospective evaluation of patients defined (iii) Risk factors analysis not well-defined

PEI = pancreatic exocrine insufficiency; US = ultrasound; BMI = body mass index.

DI = type I diabetes; DII = type II diabetes.
